# Computed tomography angiography vs 3 T black-blood cardiovascular magnetic resonance for identification of symptomatic carotid plaques

**DOI:** 10.1186/s12968-014-0084-y

**Published:** 2014-10-07

**Authors:** Jochen M Grimm, Andreas Schindler, Florian Schwarz, Clemens C Cyran, Anna Bayer-Karpinska, Tobias Freilinger, Chun Yuan, Jennifer Linn, Miguel Trelles, Maximilian F Reiser, Konstantin Nikolaou, Tobias Saam

**Affiliations:** Institute for Clinical Radiology, Ludwig-Maximilians-University Hospital Munich, Munich, Germany; Department of Medical Radiology, University Hospital and University of Lausanne, Lausanne, Switzerland; Institute for Stroke and Dementia Research, Ludwig-Maximilians-University Hospital Munich, Munich, Germany; Department of Neurology and Hertie Institute for Clinical Brain Research, University of Tuebingen, Tuebingen, Germany; Department of Radiology, University of Washington School of Medicine, Seattle, USA; Department of Neuroradiology, Ludwig-Maximilians-University Hospital Munich, Munich, Germany; Department of Radiology, University of Texas Medical Branch, Galveston, USA

**Keywords:** Plaque imaging, Ischemic stroke, Atherosclerosis, Symptomatic carotid plaque, CT, Cardiovascular magnetic resonance

## Abstract

**Background:**

The purpose of this prospective study was to perform a head-to-head comparison of the two methods most frequently used for evaluation of carotid plaque characteristics: Multi-detector Computed Tomography Angiography (MDCTA) and black-blood 3 T-cardiovascular magnetic resonance (bb-CMR) with respect to their ability to identify symptomatic carotid plaques.

**Methods:**

22 stroke unit patients with unilateral symptomatic carotid disease and >50% stenosis by duplex ultrasound underwent MDCTA and bb-CMR (TOF, pre- and post-contrast fsT1w-, and fsT2w- sequences) within 15 days of symptom onset. Both symptomatic and contralateral asymptomatic sides were evaluated. By bb-CMR, plaque morphology, composition and prevalence of complicated AHA type VI lesions (AHA-LT6) were evaluated. By MDCTA, plaque type (non-calcified, mixed, calcified), plaque density in HU and presence of ulceration and/or thrombus were evaluated. Sensitivity (SE), specificity (SP), positive and negative predictive value (PPV, NPV) were calculated using a 2-by-2-table.

**Results:**

To distinguish between symptomatic and asymptomatic plaques AHA-LT6 was the best CMR variable and presence / absence of plaque ulceration was the best CT variable, resulting in a SE, SP, PPV and NPV of 80%, 80%, 80% and 80% for AHA-LT6 as assessed by bb-CMR and 40%, 95%, 89% and 61% for plaque ulceration as assessed by MDCTA. The combined SE, SP, PPV and NPV of bb-CMR and MDCTA was 85%, 75%, 77% and 83%, respectively.

**Conclusions:**

Bb-CMR is superior to MDCTA at identifying symptomatic carotid plaques, while MDCTA offers high specificity at the cost of low sensitivity. Results were only slightly improved over bb-CMR alone when combining both techniques.

## Background

Extracranial large vessel disease is held responsible for cerebral ischemic insults in up to 20% of cases [1]. It has been shown that stenosis alone is not always a reliable marker in the assessment of a plaque’s causality for stroke [2]. There is increasing evidence that plaque morphologic aspects should be taken into consideration to assess a plaque´s vulnerability [3-5]. Accordingly, previous studies involving CT angiography or high-resolution cardiovascular magnetic resonance (CMR) have identified morphologic markers, representing plaque instability.

Using parallel imaging techniques and dedicated surface-coils 3 T high-resolution black-blood CMR (bb-CMR) is capable of reliably assessing plaque prevalence, morphology and composition, allowing lesion classification according to the modified AHA classification [6-10]. High-resolution plaque imaging with modern multi-detector CT angiography (MDCTA) delivers high spatial resolution and also allows reliable assessment of plaque prevalence, size, surface configuration and – to some extent – composition in plaques with a low grade of calcification [11,12].

Using CMR plaque imaging the plaque characteristics most closely associated with symptomatic plaques are incorporated into lesion type VI according to the modified AHA classification (AHA-LT6), comprised of its defining features ruptured fibrous cap, intraplaque hemorrhage and juxtaluminal hemorrhage/thrombus. Each of these features as well as the general classification as AHA-LT6 plaque have shown an association with ipsilateral ischemic cerebral events [13-15]. MDCTA is reported to have shown a correlation of plaque surface irregularities (e.g. ulceration), plaque density and degree of calcification with ipsilateral symptoms [16-19]. Thus, both MDCTA and CMR are able to detect differences in plaque characteristics between symptomatic and asymptomatic plaques.

The purpose of this prospective study was to perform a head-to-head comparison of MDCTA and bb-CMR with respect to their ability to identify symptomatic carotid plaques.

## Methods

This study was performed in accordance with local regulatory legislation and approved by the institutional review board. The methods used in the study were in accordance with the ethical standards laid down in the Declarations of Helsinki. This study was conducted in collaboration with the stroke unit of the Department of Neurology of the Ludwig-Maximilians-University Munich in 2008–2010. All subjects gave written informed consent.

### Patients

We examined 44 internal carotid arteries of 22 consecutive patients. Only subjects with acute ischemic stroke in the vascular area of an internal carotid artery with >50% stenosis as determined by duplex sonography were included in this study. The percentage of carotid stenosis was obtained using the NASCET method [20]. Ischemic stroke was defined as an acute lesion on diffusion weighted brain MR images (DWI) with a corresponding acute neurological deficit of more than 24 hours duration. The symptomatic artery was defined as being ipsilateral to the DWI lesion. The carotid arteries contralateral to the affected brain hemisphere served as asymptomatic control group. All patients underwent extensive clinical workup (lab, brain CMR, brain CT combined with MDCTA of the carotids, duplex sonography of the cervical arteries, 24-hour ECG, transoesophageal echocardiography) to determine the etiology of the ischemic stroke. Inclusion and exclusion criteria are summarized in Table 1.

**Table 1:** **Inclusion/Exclusion Criteria**

**Inclusion criteria**
Ischemic stroke (acute DWI^a^ lesion and corresponding acute neurological deficit of >24 h duration) in the territory of the anterior or middle cerebral artery <15 days
before both MDCTA^b^ and black-blood carotid CMR^c^
Stenosing (i.e. luminal obstruction > 50% according to NASCET^d^ criteria) atherosclerotic plaque in the internal carotid artery of the symptomatic side as determined by duplex sonography
Exclusion criteria
Stroke etiology other than large vessel disease
Bilateral infarcts on cerebral
Known contraindications against CMR or MDCTA
Allergy to contrast material
Impaired renal function (glomerular filtration rate < 30 ml/min)
Previous radiation therapy to head or neck
Surgical procedure within 24 h before bb-CMR
Previous interventional or surgical manipulation of the symptomatic carotid artery (e.g. stenting, endarterectomy)
Insufficient image quality in bb-CMR or MDCTA

^a^ DWI = diffusion-weighted imaging^b^ MDCTA = multi detector computed tomography angiography^c^ CMR = cardiovascular magnetic resonance^d^ NASCET = North American Symptomatic Carotid Endarterectomy Trial.

### Data acquisition

#### CMR

Imaging of all subjects was performed using a 3.0-T scanner (Magnetom Verio, Siemens Healthcare, Erlangen, Germany). To improve image quality, a dedicated four-channel surface carotid coil (Machnet, Elde, Netherlands) was used. The carotid coil was combined with a head coil and a head holder that prevented involuntary head movement during the scans.

Both the symptomatic and asymptomatic carotid arteries were imaged using a previously presented multi-sequence-protocol (time-of-flight (TOF), T2-, and, pre- and post-contrast T1-weighted) [21], proton density weighted images were not evaluated due to limited additional value for the present study. Best in-plane resolution was 0.5x0.5 mm^2^. Images were acquired in segments of 3.0 cm (2 mm slice-thickness x 15), centered on the carotid bifurcation. This coverage is usually sufficient to image the whole atherosclerotic carotid plaque [22]. Parallel imaging based on the generalized auto calibrating partially parallel acquisition (GRAPPA) algorithm was used for all sequences with a parallel acquisition technique (PAT) acceleration factor of 2. Post-contrast T1w images were acquired 5 minutes after injection of 0.1 mmol/kg (0.1 ml/kg) Gadolinium-DO3A-butrol (GADOVIST®, Bayer Schering, Leverkusen, Germany) over an intravenous catheter in an antecubital vein. Total scan time was 17:43 min.

### CT angiography

As stroke patient assessment is performed by several units in our clinical center, scans were performed on various CT scanners: Bright Speed S (GE Healthcare), Aquilion (Toshiba), Somatom Definition Flash (Siemens), Somatom Definitition AS + (Siemens), Sensation 64 (Siemens). All MDCTA images were obtained with coverage at least from the aortic arch to the cranium using the respective standard protocol parameters, notably a collimation of 0.625 mm or less. Non-ionic iodinated contrast material was applied intravenously adjusted to patient weight (Ultravist 370, Bayer Schering Pharma, Berlin, Germany, i.e. 0.35-0.50 g iodine per kg bodyweight at an injection rate of 4.5-6 ml/s followed by 100 ml saline at identical flow). Axial images were reconstructed to volume rendered (VR) images, which has been reported to improve depiction of carotid plaque ulceration if used additionally to axial scans [23]. Bone and soft tissue impairing the 360° view of the bifurcation were manually removed from the images. In order to prevent biases in the further reviewing process, anonymized images of the right and left carotid arteries were stored separately.

### Image review

All images were reviewed by two radiologists with 4 and 11 years of experience blinded to the patient’s clinical history. Classification was reached in consensus. Images of all patients’ left carotid arteries were reviewed first, followed by all images of the right side, in random order.

Image quality of each examination was rated on a five-point scale (1 = very poor, 2 = poor, 3 = acceptable, 4 = good, 5 = excellent) and cases with image quality <3 were excluded. All following analyses were performed on axial images.

On CMR images area-measurements of lumen, wall, and components were obtained using the image analysis tool CASCADE (University of Washington, Seattle, US). T1w images were used for obtaining values for lumen, vessel wall and total vessel area. The normalized wall index (NWI) was calculated by dividing the wall area by the total vessel area. Atherosclerotic tissue components (lipid-rich necrotic core, hemorrhage, fibrous tissue, calcification) were identified and quantified based on previously published criteria [24]. For definition of a complicated AHA-LT6 plaque according to the modified AHA classification [8], at least one of the following three criteria was required: ruptured fibrous cap, intraplaque hemorrhage or juxtaluminal hemorrhage / thrombus.

For MDCTA window settings were adjusted depending on image properties to optimize the delineation of plaque properties [19]. The relative content of calcification in the stenotic plaque (calcification / tissue <40% vs. >40%) was visually quantified and categorized. As a high grade of calcification can cause beam hardening artifacts and thus spoil measurements in adjacent tissue [25], tissue density was not measured in plaques containing calcifications >40%. MDCTA images were evaluated for presence of plaque, plaque density / plaque type, surface configuration, calcification and thrombus. Plaque type was categorized as non-calcified, mixed, and calcified. Calcification volume in mm^3^ was determined using the standard plug-in on a Syngo MultiModality Workplace (software version VE36A, Siemens Healthcare, Erlangen, Germany).

Tissue attenuation was measured in representative non-calcified plaque regions using several manually drawn ellipsoid regions of interest (ROI) per axial slice encompassing at least approximately 75% of the plaque area. Depending on plaque size, 4 to 16 ROIs with an area of up to 2 mm^2^ were drawn. During this procedure calcifications and the contrast enhanced lumen were carefully avoided. The average HU of the measured ROIs on each axial slice was recorded. From this, a mean representative HU-value for the whole plaque was calculated. Based on the mean plaque HU, plaque type was categorized as follows: non-calcified plaque associated with lipid-rich necrotic core <60 HU, mixed plaque associated with fibrous tissue 60–130 HU and calcified plaque >130 HU [26]. Plaques with calcification >40% were not measured and rated as calcified plaques. Outpouching of contrast material into or adjacent to the plaque of < 1 was considered a surface irregularity, whereas ≥1 mm was considered ulceration [27].

### Data analysis

Area measurements for each artery are given as average, minimum or maximum absolute areas, as appropriate. Plaque components are calculated as percentages of the vessel wall. Categorical variables are presented as absolute frequencies, while continuous variables are presented as mean ± [SD]. Wilcoxon’s signed-rank test was used to test differences for continuous variablesthe McNemar test was used to determine differences between categorical variables. Kruskal-Wallis test was used if more than two categories were present, e.g. degree of stenosis and of calcification. Statistical evaluation was performed using SPSS version 16.0 (IBM, Armonk, USA). A p-value of <0.05 was considered statistically significant. Sensitivity, specificity positive and negative predictive values as well as odds ratios were calculated using a 2-by-2 table.

## Results

### Patients

CT-Scans were of diagnostic quality in all subjects with a mean image quality of 4.3 [±0.65]. 20 out of 22 of the CMR examinations had an IQ ≥ 3 (90.9%) with a mean image quality of 4.2 [±0.62]. Two patients had to be excluded due to severe motion artifacts. Consequently, a total of 20 patients with 40 carotid arteries could be included and evaluated in the study. Table 2 shows general patient characteristics and cardiovascular risk factors.

**Table 2:** **Demographics** ^**a**^ **SD = Standard Deviation**

**Variable**	**Value (mean [±SD** ^**a**^ **] or N (%))**
**Age [years]**	**69.9 [±8,8]**
Male sex	15 (75%)
Body mass index [kg/m^2^]	26.0 [±2,5]
Cardiovascular risk factors	
Nicotine abuse	
Current	5 (25%)
Former	7 (35%)
Hypertension	14 (70%)
Diabetes	4 (20%)
Hypercholesterolemia	12 (60%)
Coronary artery disease	3 (15%)
Family history of cardiovascular disease	4 (20%)

### CMR

CMR detected a total of 20 AHA-LT6 plaques, of which 16 (80%) were located on the symptomatic side and 4 (20%) on the asymptomatic side. This difference was statistically significant (p < 0.001). Of AHA-LT6 plaques, a ruptured fibrous cap was found more frequently on the symptomatic than the asymptomatic side (12/20 (60%) vs. 1/20 (5%)p < 0.001) (Table 3). With respect to the degree of stenosis, AHA-LT6 plaques were found in 14 out of 19 arteries (74%) with high degree stenosis (70-99% stenosis) and in 6 out of 11 arteries (55%) with low grade stenosis (50-69%p = 0.43). No AHA-LT6 plaques were encountered in vessels with a stenosis <50%. The quantitative evaluation showed a larger normalized wall index (NWI0.88 vs. 0.78p = 0.008), and a tendency towards a smaller luminal area for symptomatic plaques compared to the asymptomatic side (6.5 ± 4.3 mm^2^ vs. 10.8 ± 7.5mm^2^p = 0.05). Plaque composition differed significantly between the symptomatic and asymptomatic side: The relative area of intraplaque hemorrhage was significantly larger on the symptomatic side (11.4 ± 17% vs. 2.1 ± 4.5%p = 0.03) (Table 3). Relative area of calcification was smaller in the symptomatic group than in the asymptomatic group (3.6 ± 5.2% vs. 7.3 ± 6.3%p = 0.03). No other statistically significant differences were found.

**Table 3:** **Plaque characteristics** ^**a**^ **NWI = normalized wall index**

	**Symptomatic side**	**Asymptomatic side**	**P-value**
**Vessel Stenosis**			
< 50% [N(%)]	0	10 (50%)	<0.001
50 – 69% [N(%)]	8 (40%)	3 (15%)	0.16
70 – 99% [N(%)]	12 (60%)	7 (35%)	0.21
**Qualitative CMR Plaque Characteristics**			
AHA Lesion Type VI [N(%)]	16 (80%)	4 (20%)	<0.001
Thin/ruptured fibrous cap [N(%)]	12 (60%)	1 (5%)	<0.001
Intraplaque hemorrhage [N(%)]	9 (45%)	4 (20%)	n.s.
Juxtaluminal hemorrhage / thrombus [N(%)]	6 (30%)	1 (5%)	n.s.
**Quantitative CMR Plaque Characteristics**			
Mean lumen area [mm^2^]	6.5 ± 4.3	10.8 ± 7.5	0.05
Mean wall area [mm^2^]	72.2 ± 28.3	64.6 ± 20.9	0.17
Mean total vessel area [mm^2^]	111.1 ± 43.4	102.3 ± 36.4	0.27
NWI^a^	0.88 ± 0.07	0.78 ± 0.11	0.008
Lipid rich necrotic core [%]	24.5 ± 12.9	14.5 ± 14	0.08
Calcification [%]	3.6 ± 5.2	7.3 ± 6.3	0.03
Intraplaque hemorrhage [%]	11.4 ± 17	2.1 ± 4.5	0.03
**MDCTA** ^**b**^ **Characteristics**			
Plaque Type			
Non-calcified [N(%)]	12 (60%)	7 (35%)	n.s.
Mixed [N(%)]	5 (25%)	10 (50%)	n.s.
Calcified [N(%)]	3 (15%)	3 (15%)	n.s.
CT Calcification Volume [mm^3^]	0.082	0.0735	n.s.

^b^ MDCTA = multi detector computed tomography angiography.

**Table 4:** **Best predictors for the symptomatic side** ^**a**^ **MDCTA = multi detector computed tomography angiography**

	**Sensitivity**	**Specificity**	**PPV**	**NPV**	**Odds ratio**
**Ulceration (MDCTA** ^**a**^ **)**	**40%**	**95%**	**89%**	**61%**	**12.7**
AHA-LT6^b^ (CMR^c^)	80%	80%	80%	80%	16.0
**Ulceration and AHA-LT6 (CTA + CMR)**	**85%**	**75%**	**77%**	**83%**	**17.0**
Thin/ruptured fibrous cap	60%	95%	92%	70%	28.5

^b^ AHA-LT6 = American Heart Association lesion type VI.

^c^ CMR = cardiovascular magnetic resonance.

**Figure 1:**
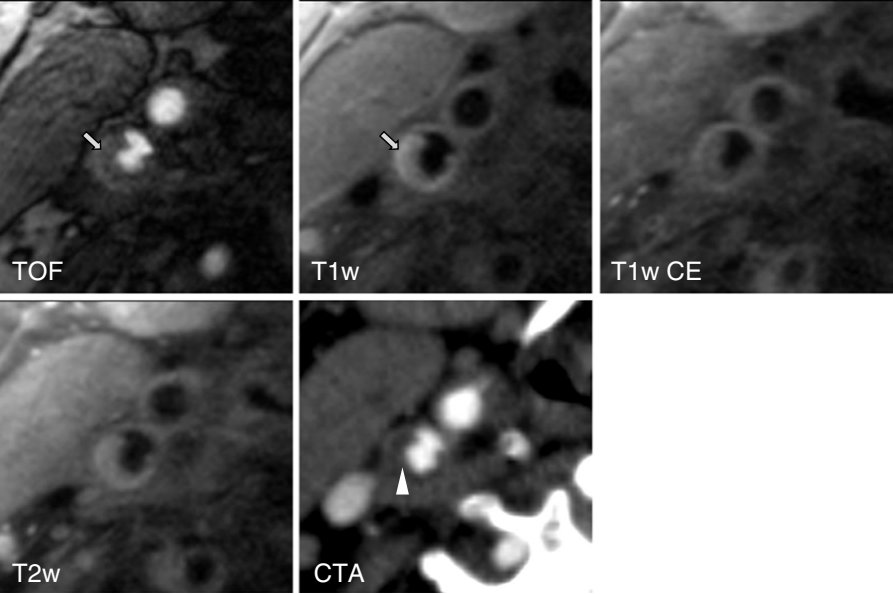
**Shows axial TOF, T1 weighted pre- and post-contrast and T2 weighted high-resolution black-blood CMR and CTA (lower right) images of an ulcerated plaque in the right internal carotid artery of an 87-year old male patient with an acute ischemic stroke in the territory of the right middle cerebral artery.** Note the clearly ulcerated plaque surface on both CMR and CTA images as well as the hypersignal of the plaque in TOF and T1 weighted images corresponding to intraplaque hemorrhage (arrow). A lack of contrast enhancement within the plaque indicates the presence of a lipid-rich necrotic core. CTA shows a non-calcified plaque with relatively hypodense plaque interior (mean plaque density = 34,5HUarrowhead).

**Figure 2:**
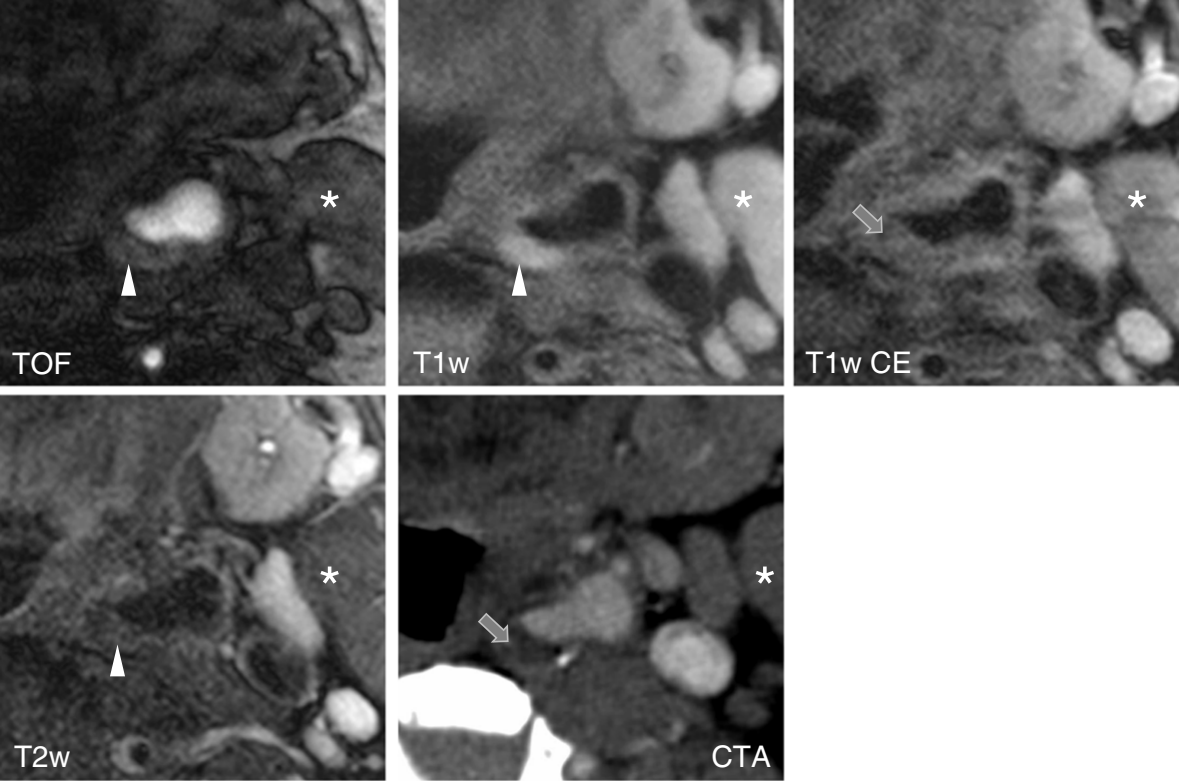
**Shows axial TOF, T1 weighted pre and post contrast and T2 weighted high-resolution black-blood CMR and CTA (lower right) images of a plaque in the left internal carotid artery of a patient with an acute ischemic stroke on the left side.** While both CMR and CT images fail to show a distinct surface defect, the fibrous cap is not entirely distinguishable and was therefore by definition classified as thin. The hyperintense signal within the plaque in TOF and T1 weighted images in combination with hypointense signal in the T2 weighted image corresponds to an intraplaque hemorrhage (arrowhead). The relative lack of contrast enhancement within the plaque indicates the presence of a lipid-rich necrotic core (arrow). Correspondingly, the CTA image shows a relatively hypodense plaque interior (mean plaque density = 65,1 HU). The hyperdense area in the dorsal wall of the plaque corresponds to a hypointense signal in the MR images and is consistent with a marginal calcification. * Sternocleid muscle

**Figure 3:**
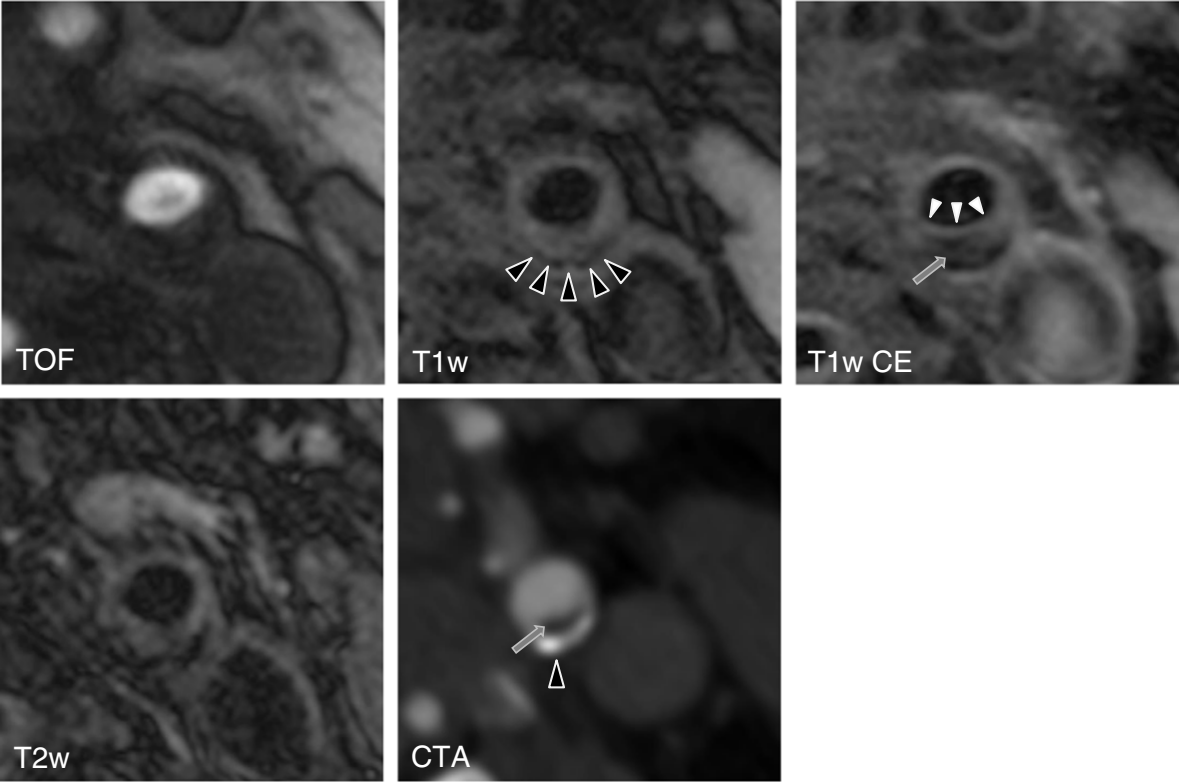
**Shows axial TOF, T1 weighted pre- and post- contrast and T2 weighted high-resolution black-blood CMR and CTA (lower right) images of a stable carotid plaque on the asymptomatic left side in a 66 year old patient who had suffered from right hemispheric stroke.** Both CMR and CTA images show the presence of an AHA lesion type 7 plaque in the dorsal wall of the left proximal internal carotid artery. After administration of contrast material the thick fibrous cap is delineated as a hyperintense rim in the T1 weighted contrast enhanced images, separating the plaque from the lumen (white arrowheads). Also note the hypointense signal of the plaque interior in the T1 weighted contrast enhanced image as well as the hypodense area in the CTA image corresponding to a large lipid-rich necrotic core (arrow), measured at 166 HU, probably due to blooming artifacts caused by its calcified portion. The hypointense rim in the peripheral plaque in all MR sequences and the corresponding hyperdense area in the CTA image indicate the presence of a calcification (black arrowheads).

Figures 1, 2, and 3 show black-blood CMR and MDCTA imaging examples of symptomatic (Figures 1 and 2) and asymptomatic (Figure 3) carotid plaques.

### Computed tomography

#### Vessels / plaque

Plaques were detected in all symptomatic vessels (20/20, 100%), while two vessels of the asymptomatic side showed no sign of atherosclerosis (18/20, 90%p = 0.49). Plaque types on the symptomatic vs. asymptomatic side as detected by CT were distributed as follows: non-calcified plaque 12 (60%) vs. 7 (35%p = 0.21) mixed plaque 5 (25%) vs. 10 (50%p = 0.19) and calcified plaque 3 (15%) vs. 3 (15%p = 1) (Table 3). Calcification volumes were not significantly different between the symptomatic (0.0820 ± 0.159 mm^3^) and the asymptomatic (0.0735 ± 0.077 mm^3^) side (p = 0.22). Degrees of stenosis on the symptomatic vs. asymptomatic side according to the NASCET criteria were distributed as follows: <50% stenosis 0 (0%) vs. 10 (50%p < 0.001), 50-69% stenosis 8 (40%) vs. 3 (15%p = 0.16) and 70-99% stenosis 12 (60%) vs. 7 (35%p = 0.21). Kruskal-Wallis test for degree of stenosis yielded an adjusted H of 13.2, corresponding to a p-value of <0.001. Differences between groups were calculated using the McNemar test (Table 3). Stenosis <50% was only encountered in asymptomatic carotids. None of the vessels were occluded.

### Surface

CT showed 8 vessels with ulceration on the symptomatic side (40%) and one (5%) in an asymptomatic internal carotid artery (p = 0.005). Multiple ulcerations within one vessel were not observed. In one case we found ulcers in both carotid arteries. The proportion of ulcerations in all arteries was not significantly different between 50-69% stenoses (3 out of 1127%) compared to stenoses of 70-99% (6 out of 1932%p = 1).

### Tissue density and calcification

Symptomatic plaques had a significantly lower density compared to asymptomatic plaques (48.9 ± 15.6 HU vs. 61.8 ± 15.6 HUp = 0.046). Kruskal-Wallis and McNemar tests revealed no significant differences between symptomatic and asymptomatic plaques regarding presence of calcification. Calcification volume was not significantly different between symptomatic and asymptomatic plaques.

### Thrombus

Using MDCTA we could observe the ‘donut sign’ - a filling defect within the lumen completely surrounded by contrast media indicating presence of a thrombus - in only one of the 20 symptomatic carotid arteries (5%) versus none in the asymptomatic arteries (0%).

### Best predictors

AHA-LT6 as single predictor for the symptomatic side resulted in a sensitivity, specificity, PPV and a NPV of 80%, 80%, 80% and 80%, respectively, yielding an accuracy of 80% and an odds ratio of 16.0 (95% confidence interval: 2.8-108.9). With CMR 4 out of 20 symptomatic plaques were not predicted correctly. In one of these 4 patients, MDCTA was able to detect an ulcerated plaque.

When a thin or ruptured fibrous cap was used as single predictor, sensitivity, specificity, PPV and NPV were 60%, 95%, 92% and 70%, respectively. The accuracy was calculated at 77.5%the odds ratio was 28.5 (95% confidence interval 3.2-257.5).

Using ulceration in MDCTA as single predictor for the symptomatic side led to a sensitivity, specificity, PPV and a NPV of 40%, 95%, 89% and 61%, accuracy of 67.5% and an odds ratio of 12.7 (95% confidence interval: 1.3-306).

Ulcer as detected in CT combined with AHA-LT6 Plaque in CMR led to a sensitivity of 85%, specificity of 75%, a PPV of 77% and a NPV of 83%, yielding an accuracy of 80% and an odds ratio of 17.0 (95% confidence interval: 2.8-121.2Table 4).

**Figure 4:**
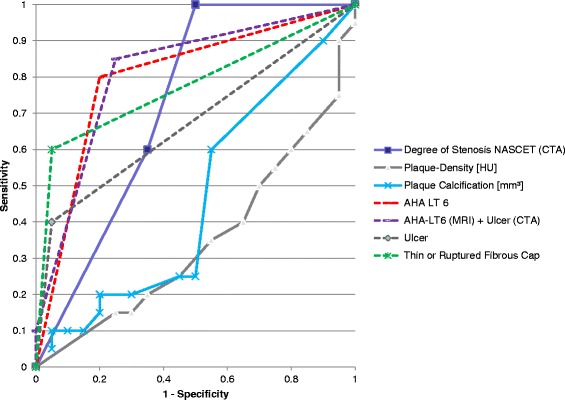
**Shows the ROC graph of various variables.** Lines are dotted where only one value was present. Note that especially AHA-LT6 with and without ulcer in CTA as well as thin or ruptured fibrous cap show high sensitivity and specificity, while plaque density in HU and volume of calcification are not suitable as predictors of the symptomatic side.

The ROC graph in Figure 4 gives an overview over the presented variables and their respective sensitivity and specificity.

## Discussion

This study shows that both MDCTA and bb-CMR of the carotid arteries are able to detect differences between symptomatic and asymptomatic plaques in patients with ischemic large vessel stroke. CMR using AHA-LT6 as single criterion showed higher sensitivity, negative predictive value and accuracy than ulceration as the best MDCTA variable while the latter delivered a better specificity and positive predictive value but suffered from a low sensitivity. Even better specificity and positive predictive values were found when using a thin or ruptured fibrous cap as detected on bb-CMR as single predictor. Prediction of the symptomatic side was minimally enhanced when information from both MDCTA and CMR were combined.

### AHA-LT6

Complicated AHA-LT6 plaques were more frequently encountered in symptomatic than in asymptomatic arteries (80% vs. 20%, p < 0.001). Consequently type III, IV, V and VII, which reflect clinically more stable plaques, were more common in asymptomatic vessels. These results are in line with findings of Saam et al. who found a statistically significant association between complicated AHA-LT6 carotid plaques and ipsilateral symptoms in a cohort of 23 symptomatic patients who underwent CMR-plaque imaging [13]. This makes AHA-LT6 a useful parameter for risk assessment in atherosclerotic carotid plaques. In our study, as a single criterion, the presence of an AHA-LT6 plaque proved to be the best overall predictor for the symptomatic side.

We noted a greater frequency of AHA-LT6 plaques in high degree stenoses compared to low degree stenoses. Even though not statistically significant, this result is in line with findings of previous studies [28,29], which report an increasing prevalence of vulnerable plaques with increasing degree of stenosis.

### Ulceration

Former studies could show that ulceration of the carotid arteries is associated with cerebral lesions and is encountered more frequently in symptomatic patients [17,30-32]. The present study found ulcerations with a prevalence of 40% in symptomatic carotid arteries and in 5% of the asymptomatic arteries. Overall prevalence of ulceration was 32% in high grade stenoses (70-99%), 27% in low grade stenoses (50-69%) and 0% in non-stenosed arteries. The reported prevalence of plaque ulceration in our study is similar to the NASCET study, which reported ulcerations in 35% of vessels with a stenosis >70% as detected by DSA [33].

In our study, using ulceration in MDCTA as the sole predictor for the symptomatic side led to a relatively low sensitivity but a high specificity. The lower sensitivity may be explained by the fact that other mechanisms that are hard to detect in MDCTA, such as rupture of the fibrous cap or intraplaque hemorrhage potentially followed by thrombus formation and consequent embolisation may also cause cerebrovascular incidents.

### Other factors

While other plaque properties analyzed in this study failed to reach the predictive power of AHA-LT6 or plaque ulceration, they may still be useful for plaque characterization and general risk assessment.

Analysis of the plaque density showed that plaques on the symptomatic side had significantly lower HU values than asymptomatic plaques. This may be due to higher lipid content and confirms the findings of other studies by Saba et al. [30] and Serfaty et al. [25] which both found a statistically significant association between fatty plaque or decreased plaque density and neurological symptoms in cohorts of 112 and 141 patients. Trelles et al. similarly found a greater thickness of the non-calcified plaque component in AHA-LT6 plaques in a study on 51 stroke patients [34]. These results are also in line with previous observations in coronary plaques which suggested that plaques with a large lipid-rich/necrotic core strongly correlate with formation of thrombi and plaque disruption [35]. Despite these differences in HU, a reliable cut-off point to distinguish symptomatic from asymptomatic plaques has not yet been determined [19].

Juxtaluminal hemorrhage / thrombus impose a high risk of distal embolization and thus thrombotic occlusion of a distal vessel. In a previous histological study with 241 patients Fisher et al. observed that thrombus is highly associated with ipsilateral symptoms and plaque ulceration (p < 0.005) [33]. Using MDCTA we could identify a fresh thrombus (“donut sign”) in only one carotid artery (2.5%). CMR, however, showed juxtaluminal hemorrhage / thrombus in 30% of the symptomatic and 5% of the asymptomatic arteries. Our study thus shows that for detection of juxtaluminal hemorrhage / thrombus CMR is more sensitive than MDCTA, a fact that may be attributed to MDCTA’s comparatively poor soft tissue contrast and its inability to identify the fibrous cap and intraplaque hemorrhage. In 6 out of 7 cases thrombi detected by CMR were associated with a rupture of the fibrous cap. This supports the thesis that thrombi evolve from surface defects of plaques presenting thrombogenic components.

In our study a thin or ruptured fibrous cap itself was closely associated with the symptomatic side (OR 28.5). This may be attributed to thrombogenic potency of surface defects of the fibrous cap with subsequent formation of unstable thrombi, potentially causing embolism. Our results indicate that a thin or ruptured fibrous cap may be a more accurate predictor than juxtaluminal hemorrhage/thrombus. Compared to AHA-LT6, a thin or ruptured fibrous cap as single predictor showed superior specificity and positive predictive value, while sensitivity and negative predictive value were inferior. Because of its high specificity and positive predictive value a thin or ruptured fibrous cap may be a factor to be considered separately.

### Bb-CMR or MDCTA?

Based on the data of our study bb-CMR is better suited than MDCTA to identify symptomatic carotid plaques. This is mainly owed to the better detection of subtle plaque features on bb-CMR. In order to further assess the potential of bb-CMR, several prospective studies, such as the CAPIAS trial (NCT01284933) [36] or the PARISK study (NCT01208025) are currently under way which examine the ability of carotid bb-CMR to predict recurrence of ischemic stroke in patients with less than 70% stenosis.

While the diagnostic value of bb-CMR regarding the characterization of carotid plaques cannot reasonably be disputed, it is still not routinely applied in many centers. This may be attributed to a generally lower availability of suitable CMR equipment and longer scan times of bb-CMR compared to MDCTA, requiring a higher degree of patient cooperation. Another limiting factor for carotid CMR in the acute setting is the narrow time window for treating acute strokes, so examination times need to be kept reasonably short. It needs to be noted that the use of a dedicated carotid coil, which is not available at every center, enhances MR image quality. However, with careful design of the examination and its sequence parameters, image quality should be sufficient to determine the major plaque features even without such a dedicated coil.

On the other hand, MDCTA requires application of a considerable amount of ionizing radiation and, more importantly, iodinated contrast agent with the known adverse effects, especially in the patient population with cardiovascular risk factors. Logistically, bb-CMR of the carotids can be performed immediately following brain CMR with reasonable extension of the examination time, which will at least in part be compensated by avoiding a transfer of the patient between the CT and CMR device.

### Limitations

One limitation of this study is the use of different CT scanners, so that examination parameters are not completely identical. We chose to include examinations from various CT scanners to increase the number of patients and accounted for possible differences in image acquisition parameters by applying a uniform image quality score to all images.

Furthermore, our study followed a cross sectional design by examining patients who were already symptomatic. To assess the predictive value of different plaque characteristics regarding the risk of future cerebrovascular events, longitudinal studies are necessary. It should be noted that our study population contains a comparatively large number of vessels with high degree stenoses. This is owed to the inclusion criteria requiring ≥50% stenosis in the artery ipsilateral to the ischemic stroke and does not represent the prevalence of high degree stenosis in the general population. It needs to be noted that we performed all tests on subjects of a symptomatic population where by definition the prevalence of symptomatic carotid plaques is 100%. This may influence the absolute validity of sensitivity, specificity, positive and negative predictive values for the reported plaque features at predicting the symptomatic side. It should, however, not significantly impair the comparability of the different predictors with each other.

3D sequences were not part of this study. While these might have increased the arterial coverage or decreased examination times, we chose to not apply them in this study because they are less well validated and may provide less spatial in-plane resolution. Furthermore, coverage of the 2D sequences used was sufficient to depict the entire plaque in each case so that it seemed safe to use the previously established 2D protocol, which has proven to deliver excellent image quality while being robust and reliable.

We did not use contrast enhanced MR angiography (CEMRA) because we performed perfusion imaging of the plaques, which was not evaluated for this study. Evaluation of CEMRA might have increased the sensitivity of CMR to detect surface irregularities in carotid plaques. However, several studies have shown that ulcerations are an unreliable predictor of patient’s symptoms and we therefore believe that while CEMRA might have further accentuated the superiority of CMR, this would not have significantly changed our conclusion.

## Conclusions

Bb-CMR is superior to MDCTA at detecting symptomatic plaques. Predictive power of CMR using AHA-LT6 is only slightly enhanced if combined with ulceration from MDCTA. Presence of a thin or ruptured fibrous cap on MR images may be a useful individual property for predicting the presence of symptoms due to its excellent specificity and positive predictive value. Although MDCTA has a lower sensitivity to identify the symptomatic side, it does offer some insights into plaque vulnerability and remains useful in patients who cannot undergo CMR.
